# Population-Wide Associations between Common Viral Pathogens and Self-Reported Arthritis: NHANES 2009-2012

**DOI:** 10.1155/2018/7684942

**Published:** 2018-10-01

**Authors:** Anna Shmagel, Grace Skemp-Dymond, Lisa Langsetmo, John T. Schousboe, Kristine Ensrud, Robert Foley

**Affiliations:** ^1^Assistant Professor of Medicine in the Division of Rheumatic and Autoimmune Diseases at the University of Minnesota, Division of Rheumatic and Autoimmune Diseases, 420 Delaware Street SE, MMC 108, Minneapolis, MN 55455, USA; ^2^General Internist at the Center for Outpatient Care in Edina, Minnesota, USA; ^3^Senior Research Associate for Epidemiology and Community Health at the University of Minnesota, USA; ^4^Rheumatologist, Department of Rheumatology, Park Nicollet Clinic and HealthPartners Institute, Bloomington, Minnesota, USA; ^5^Assistant Professor, Division of Health Policy and Management at the University of Minnesota, USA; ^6^Professor of Medicine and Epidemiology & Community Health, University of Minnesota, Core Investigator at the Minneapolis VA Center for Chronic Disease Outcomes Research, USA; ^7^Associate Professor of Medicine at the University of Minnesota, Division of Renal Diseases and Hypertension, USA

## Abstract

**Objective:**

Persistent infectious agents have been implicated in chronic and recurrent inflammation, which may trigger or worsen many types of arthritis. Our objective was to determine whether exposure to herpes simplex virus (HSV) and human papillomavirus (HPV) is associated with self-reported arthritis among US adults.

**Methods:**

We used data from two consecutive cycles of the National Health and Nutrition Examination Survey (NHANES) from 2009 until 2012 (N of examined adults ages 20-69 = 9483). Participants were classified as having arthritis by self-report. Viral serology for HSV-1 and HSV-2 and HPV PCR studies from oral rinse and vaginal swabs were available for analysis. We compared HSV-1 and HSV-2 seropositivity as well as oral and vaginal HPV DNA positivity between participants with self-reported arthritis vs. those without, adjusting for age, gender, race, income, education, BMI, and the use of immunosuppressive medications. We used three comparator outcomes, gout, kidney stones, and hypertension, to evaluate whether the associations were specific or not to arthritis.

**Results:**

Arthritis was associated with older age, female gender, non-Hispanic White and Non-Hispanic Black race, higher BMI, and lower socioeconomic status. HSV-2 seropositivity, but not HSV-1 seropositivity, was independently associated with arthritis after adjustment for age, gender, race, income, education, BMI, and the use of immunosuppressive medications: AOR 1.48 (1.10-1.99). Oral HPV DNA positivity was also independently associated with arthritis: AOR 1.63 (1.17-2.28). After adjustment, there was no statistically significant difference in vaginal HPV DNA positivity between those with vs. those without arthritis: AOR 1.22 (0.90-1.66). There were no significant associations between viral exposures and any of the comparator outcomes.

**Conclusions:**

HSV-2 seropositivity and oral HPV DNA positivity were associated with self-reported arthritis and not with comparator outcomes, after adjustment for multiple potential confounders. These findings should be confirmed in longitudinal studies.

## 1. Introduction

The pathogenesis of many rheumatic diseases is classically described by a model in which genetic predisposition and exposure to an environmental trigger lead to the development of disease phenotype. Several environmental triggers have been explored in association with arthritis, with a recent focus on infectious agents [1]. Viral pathogens have been implicated in the development of infectious arthritis (Parvovirus B19, Chikungunya/Alphaviruses, Hepatitis C Virus, HTLV1, Rubella), as well as rheumatoid arthritis (Epstein-Barr Virus, Cytomegalovirus, Human Endogenous Retrovirus, and herpesviruses 6 and 8), psoriatic, and reactive arthritis (HIV).

Members of the herpesviruses family, including EBV and CMV, are known for the ability to establish latent infections. EBV and CMV have emerged as viruses of interest in the pathogenesis of autoimmune joint disease, seen in rheumatoid arthritis, Sjogren's syndrome, and SLE by creating a sustained immune response and potentiating the chance of molecular mimicry [2–6]. More recently, chronic inflammation has also been implicated in the pathogenesis of osteoarthritis [7]. While less evidence is available for HSV and HPV's role in autoimmune disease, these pathogens are likely candidates for inducing chronic arthritis as they too produce latent infections, continuously stimulate the immune system, and cause ongoing low-grade systemic inflammation [8].

The intent of our study was to assess further whether exposure to herpesviruses (HSV-1, HSV-2) and human papillomaviruses (HPV) is associated with self-reported arthritis among community-dwelling adults. These pathogens are plausible triggers of chronic arthritis, as they are both common and persistent. HSV-1 and HSV-2 affect 60% and 20% of US adults, respectively [9]. After exposure, herpesviruses persist indefinitely in the majority of hosts [10]. HPV virus infections, while also very common, are usually cleared within six months to two years. However re-exposure is frequent and HPV persistence has been associated with development of chronic disease, such as cervical cancer [11]. In theory, chronic viral infections may be triggering maladaptive immune responses and low-grade systemic inflammation leading to cartilage degradation and periarticular bone changes. In addition, people with chronic low-grade infections may have an amplified pain response [12]. We tested the hypothesized association between HSV-1, 2, HPV, and self-reported arthritis in a cross-sectional population-based study.

## 2. Materials and Methods

The National Health and Nutrition Examination Survey (NHANES) is a biannual cross-sectional research survey conducted by the National Center for Health Statistics (NCHS) to assess the health and nutritional status of the US population. It combines household interviews, physical examinations, and a variety of laboratory tests, selected on the basis of public health significance [13]. The NHANES design permits sample weights, allowing estimates to be made that are generalizable to the US population [14]. We used data from two consecutive cycles of the National Health and Nutrition Examination Survey (NHANES) from 2009 until 2012 (most recent available cycles with full data, N of examined adults ages 20-69 = 9483). Participants were classified as having arthritis by self-report on the medical conditions survey administered during the household interview. Participants were asked whether a doctor has ever told them they had arthritis. Answering “Yes” was classified as having arthritis. Self-reported gout, kidney stones, and hypertension from the same survey were used as comparator outcomes, given their established metabolic etiology, relatively high prevalence, and unlikely association with viral infections.

Demographic information was collected during the household interview. History of sexually transmitted diseases (STDs) was obtained via a computer-assisted self-interview in a private setting. Full data on STDs was available on three consecutive NHANES cycles at the time of study (2009-2014). All available data was used in the STD analysis. BMI was calculated from standardized measurements obtained during the physical exam component, and the use of immunosuppressive medications was assessed from participants' report and confirmed with available pill bottles (full list in Appendix B).

Viral studies were collected during the mobile examination center (MEC) component of the study. Solid-phase enzymatic immunodot assays were used to detect antibodies reactive to glycoprotein gG-1 for HSV-1 and glycoprotein gG-2 for HSV-2, respectively. HSV-1 and HSV-2 serologies were classified as positive or negative, and available on all participants ages 20 to 49. Oral HPV was obtained from oral rinse on 92% of participants ages 20 to 69. All oral samples were reported as collected and the missing data was entirely due to available samples not evaluated. HPV DNA was purified according to the Puregene DNA purification kit protocol and analyzed by means of a multiplex polymerase-chain-reaction (PCR) assay (Roche Molecular Systems, Inc., Alameda, CA). Vaginal HPV swabs were self-collected by female study participants ages 20 to 59, and 88% were available for analysis. Attrition for vaginal samples was due to inadequate samples or participant refusal. HPV DNA was extracted and analyzed by Digene Hybrid Capture (hc2) and Roche Linear Array. Results were classified as positive or negative.

We compared HSV-1 and HSV-2 seropositivity, as well as HPV DNA positivity between those with arthritis and without, using Chi-square tests. Logistic regression models were used to estimate the associations of these primary predictors with the primary outcome (arthritis) and each of the comparator outcomes (gout, kidney stones, and hypertension), adjusted for age, gender, race, income, education, BMI, and the use of immunosuppressive medications. Additionally, sensitivity analyses were performed excluding subjects who reported immunosuppressive medication use (Appendix B). In accordance with NHANES methodology, each survey participant represents a specific proportion of the US population; hence all results were expressed in weighted percentages of the US population. Primary sample unit (“sdmvpsu”) and stratum (“sdmvstra”) variables, as well as adjusted 4-year interview weights, were used to obtain national estimates for questionnaire variables, and adjusted 4-year mobile examination center (MEC) weights, for MEC variables. 6-year MEC weights were used for self-reported STD analyses. A 95% confidence level was set for all tests of significance. All statistical analyses were performed in SAS 9.4 (SAS Institute, Inc., Cary, NC).

## 3. Results 

**Figure 1:**
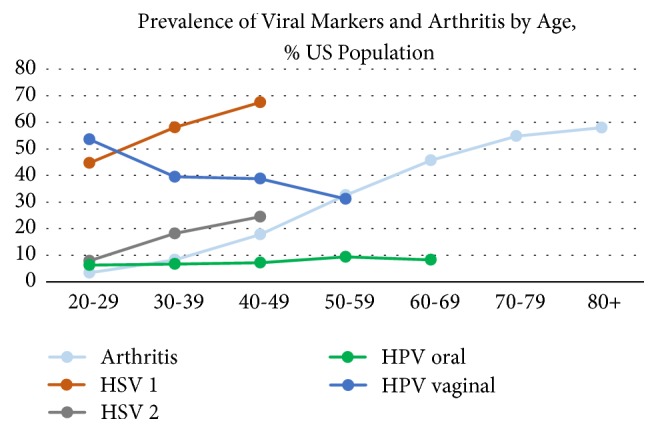
The prevalence of positive viral markers and arthritis by age in the US population, NHANES 2009-2012. Viral studies were collected on the full study sample with pre-determined age cutoffs: HSV-1 and HSV-2 serologies: ages 20 to 49. Oral rinse for HPV DNA, ages 20 to 69, vaginal swabs for HPV DNA, ages 20 to 59. The prevalence of self-reported arthritis and viral markers in the population varied with age. Self-reported arthritis prevalence increased with age, from 3.4% in the 2^nd^ decade of life to 41.9% in the 6^th^ decade of life, which was the age range of this study (chi-square p value <0.001). HSV-1 and HSV-2 seropositivity increased with age (p <0.001) and oral HPV DNA positivity increased gradually up to the 5^th^ decade of life and then plateaued (p = 0.045). Vaginal HPV DNA positivity declined with age (p<0.001).

The overall estimated population prevalence of self-reported arthritis among those aged 20-69 years in the US was 19.1% (0.7). Arthritis was more common among women vs. men, older vs. younger adults, non-Hispanic Whites vs. other race/ethnicities, and those with higher vs. lower BMI (Table 1). Among those who reported having arthritis, 3.2% (0.5) were taking immunosuppressive medications, which was higher than those who did not report arthritis: 0.6% (0.1), p<0.001, and AOR 4.73 (2.64-8.46). The prevalence of viral markers in the population varied with age (Figure 1). HSV-1 and HSV-2 seropositivity increased with age, vaginal HPV DNA positivity declined, and oral HPV DNA positivity did not significantly change.

The association between self-reported arthritis and viral markers is displayed in Table 2. HSV-1 seropositivity was more frequent among those with arthritis than without arthritis (69.1% vs 56.0%, p<0.001) as was HSV-2 seropositivity (31.2% vs 15.7%, p<0.001). After adjustment for age, gender, race, income, education, BMI, and the use of immunosuppressive medications, HSV-2, but not HSV-1 seropositivity, was significantly associated with arthritis with: AOR 1.48 (1.10-1.99) for HSV-2 and 1.25 (0.96-1.62) for HSV-1.

Oral HPV DNA was positive in 10.3% of those with arthritis vs 6.9% of those without (p=0.003), and vaginal HPV DNA, in 42.1% vs 40.4%, respectively (p=0.65). Oral HPV DNA positivity was associated with arthritis after adjustment for age, gender, race, income, education, BMI, and the use of immunosuppressive medications: AOR 1.63 (1.17-2.28). There was no statistically significant association for vaginal HPV DNA positivity: AOR 1.22 (0.90-1.66). Full logistic regression models are available in Appendix A.

We also evaluated the associations between self-reported STDs and arthritis (Table 2). We found positive associations between self-reported history of genital herpes and arthritis: AOR 1.42 (1.02-1.99), as well as history of genital warts and arthritis: AOR 1.56 (1.11-2.21). History of Chlamydia was not significantly associated with arthritis: AOR 1.17 (0.50-2.73). Gonorrhea was inversely associated with arthritis: 0.26 (0.08-0.87).

We used comparator outcomes (gout, kidney stones, and hypertension) to evaluate whether the association between the viruses of interest and arthritis was merely an association with general morbidity/chronic illness. The prevalence of gout in US adults ages 20-69 was 2.7% (0.3), kidney stones was 7.8% (0.4), and hypertension was 26.0% (0.8). By unadjusted estimates, there was a numerically higher prevalence of HSV-1 seropositivity among subjects with kidney stones and hypertension, and HSV-2 seropositivity, among subjects with hypertension. However, these numeric differences were attributed to common risk factors: older age, non-Hispanic race, higher BMI, and lower education level (data not shown). None of the comparator outcomes were significantly associated with HSV-1, HSV-2, or HPV after adjustment for age, gender, race, income, education, BMI, and the use of immunosuppressive medications (Table 3). We also stratified HSV and HPV prevalence by self-reported type of arthritis (Appendix B); however, precise identification of the nature of arthritis was not feasible in this study.

In summary, after adjustment for demographic characteristics, BMI, and the use of immunosuppressive medications, HSV-2 seropositivity was associated with self-reported arthritis in US adults ages 20-49; HPV DNA positivity in oral samples was associated with self-reported arthritis in US adults ages 20-69, but vaginal HPV DNA positivity was not associated with arthritis in women ages 20-59. History of genital herpes and genital warts were also associated with arthritis. Positive viral studies were not associated with any of the comparator outcomes (gout, kidney stones, or hypertension).

## 4. Discussion

We demonstrate that young and middle-aged US adults with self-reported arthritis have an elevated prevalence of HSV-2 seropositivity and oral HPV DNA positivity. These results from a population-based study suggest several intriguing hypotheses about the relationship between these viral infections and arthritis.

While limited evidence is available for HSV and HPV's role in arthritis, these pathogens are likely candidates for inducing chronic arthritis as they produce chronic and latent infections, continuously stimulate the immune system, and cause ongoing low-grade systemic inflammation [15]. In animal models, HSV induced both acute inflammatory arthritis, persistent synovitis, lymphocytic infiltration, and cartilage erosion in the joints in the absence of active viral replication [8]. In addition to initiating chronic inflammation with infection, there exists an intriguing theory that HPV may act as an autoantigen in rheumatoid arthritis. The HPV-47 E2 protein was found to be homologous to profilaggrin 306-324. Elevated levels of antibodies against citrullinated profilaggrin 306-324 are found in the serum of patients with rheumatoid arthritis and may be associated with higher disease activity and radiographic progression [16].

Another possible explanation for our findings is that HSV-2 and HPV are associated with chronic musculoskeletal pain rather than true arthritis. HSV-1 and 2 are neurotropic viruses, and can directly activate glial cells [17]. When activated, glial cells release pain transmitters and substances that stimulate pain-responsive neurons [18]. Local or systemic immune response to an infection can induce and sustain neuropathic pain via inflammatory cytokines and chemokines [19, 20]. Additionally, oxidative stress mediators, such as F2-isoprostane, can be induced by HSV and are associated with persistent oxidative damage to the brain tissue after the active phase of HSV infection in mice [21]. A positive correlation with F2-isoprostane and joint pain severity has also been observed in humans [22]. Finally, the cortisol axis may play a role in the association between HSV-2 and chronic pain, as HSV-2 preferentially infects neurons that express glucocorticoid and adrenergic-beta1 receptors, while HSV-1 does not [23]. Persistent stress and anxiety have been associated with more frequent genital herpes reactivations, and are also closely intertwined with chronic pain [24, 25].

Our study's strengths include a large nationally representative sample with standardized questionnaires and lab collection methods. Several limitations, however, must be considered. NHANES is a cross-sectional survey, with many self-report variables that are subject to measurement error and recall bias. Outcome misclassification bias needs to be weighed as arthritis was self-reported. Several studies demonstrated that misclassification of arthritis by self-report is frequent, with percent agreement ranging between 43% and 96%, varying between different types of arthritis [26, 27]. Misclassification of the outcome tends to bias results toward the null and decrease precision, possibly leading to overly conservative estimates in our study. Unfortunately, we could not reliably differentiate between specific types of arthritis by self-report. A more granular arthritis outcome measure is needed to make any strong conclusions. Temporal relationships and causality cannot be established from this study design, and, as with any observational study, residual confounding is likely present. Viral studies were generally available on younger participants, and important data in the older population may be missing. Results should not be extrapolated on younger and older subjects. The study was also underpowered to evaluate associations between specific types of HPV and arthritis.

In conclusion, this cross-sectional population-based study suggests an association between HSV-2 seropositivity and self-reported arthritis, as well as oral HPV DNA positivity and self-reported arthritis, adding to the growing body of evidence that links infections with chronic musculoskeletal symptoms. Despite a large population-based sample with uniform measurement of viral markers, characterization of arthritis was limited in our study. Different research models should be pursued to further evaluate the nature of association between viral infections and specific types of arthritis to facilitate the development of effective risk reduction and novel treatments for arthritic conditions

## Figures and Tables

**Table 1:** Demographic characteristics of US adults, ages 20-69, with and without arthritis (N = 9483).

	%** with arthritis (SE**%**)**	%** w/o arthritis (SE**%**)**	**Chi-Sq ** **P-value**	**Adjusted OR ** **(95% CI)**	**AOR ** **P-value**
	**N=1905**	**N=7578**			
**Age ** ( yrs)					
20-29	3.6 (0.3)	26.0 (1.1)	<0.001	1 (Ref.)	0.002
30-39	8.2 (0.6)	23.1 (0.6)		1.40 (0.98-1.99)	
40-49	19.0 (1.0)	22.4 (0.7)		1.69 (1.01-2.83)	
50-59	34.1 (1.4)	17.9 (0.6)		1.89 (0.96-3.71)	
60-69	35.0 (1.3)	10.6 (0.5)		1.66 (0.67-4.08)	
**Women**	59.5 (0.9)	48.9 (0.5)	<0.001	1.56 (1.40-1.74)	<0.001
**Race**					
Non-Hispanic Black	11.7 (1.2)	11.9 (1.0)	<0.001	1 (Ref.)	<0.001
Non-Hispanic White	74.6 (1.9)	62.4 (2.2)		1.23 (1.03-1.48)	
Hispanic	9.3 (1.2)	16.8 (1.7)		0.66 (0.56-0.77)	
Other	4.4 (0.6)	8.9 (0.7)		0.61 (0.48-0.78)	
**Education**					
College and higher	17.7 (1.3)	15.4 (0.8)	<0.001	1 (Ref.)	<0.001
GED/AA degree	56.8 (1.4)	52.8 (1.0)		1.46 (1.18-1.80)	
Less than High school	25.5 (1.5)	31.8 (1.2)		1.38 (1.16-1.65)	
**Annual HH income **$					
65 000 and higher	38.1 (2.1)	44.3 (1.6)	<0.001	1 (Ref.)	0.1
20-64 000	43.6 (1.7)	42.1 (1.1)		1.39 (0.93-2.06)	
<20 000	18.3 (1.5)	13.6 (0.8)		1.11 (0.83-1.47)	
**BMI**					
<25	20.9 (1.1)	33.7 (1.0)	<0.001	1 (Ref.)	<0.001
25-30	28.4 (1.5)	33.6 (0.7)		1.13 (0.92-1.37)	
>30	50.7 (1.4)	32.8 (0.7)		2.07 (1.77-2.41)	
**Immunosuppressive medication use**	5.3 (0.7)	0.9 (0.1)	<0.001	4.73 (2.64-8.46)	<0.001

SE: standard error. Chi-Sq: Chi-square. OR: odds ratio. AOR: adjusted odds ratio, adjusted for age, race, gender, and education. CI: confidence interval. GED: general educational development. AA: associate's. HH: household. BMI: body mass index. Reference category not displayed for binary variables. All estimates are weighted to represent the national population.

**Table 2:** Associations of viral markers and sexually transmitted diseases with self-reported arthritis, US adults ages 20-69 (N = 9483).

	%** with arthritis (SE%)**	%**w/o arthritis (SE**%**)**	**OR (95**%** CI)**	**OR ** **P-value**	**AOR (95**%** CI)**	**AOR** **p-value**
**Viral Markers**						
HSV 1 ab (N=5438)	69.1 (2.4)	56.0 (1.4)	1.68 (1.34-2.12)	<0.001	1.25 (0.96-1.62)	0.09
HSV 2 ab (N=5438)	31.2 (2.2)	15.7 (0.7)	2.28 (1.82-2.84)	<0.001	1.48 (1.10-1.99)	0.009
HPV oral PCR (N=8678)	10.3 (1.2)	6.9 (0.5)	1.55 (1.15-2.09)	0.004	1.63 (1.17-2.28)	0.004
HPV vaginal PCR (N=3469)	42.1 (3.5)	40.4 (1.3)	1.07 (0.80-1.43)	0.6	1.22 (0.90-1.66)	0.2
**History of STDs**						
HPV (women) (N=4773)	15.1 (2.4)	20.0 (0.9)	0.71 (0.49-1.03)	0.07	1.28 (0.82-1.99)	0.3
Genital herpes (N=9443)	24.2 (3.1)	16.0 (0.6)	1.67 (1.20-2.34)	0.003	1.42 (1.02-1.99)	0.04
Genital warts (N=9450)	25.5 (2.8)	15.9 (0.6)	1.81 (1.30-2.51)	<0.001	1.56 (1.11-2.21)	0.01
Gonorrhea (N=9446)	4.6 (3.3)	16.4 (0.6)	0.25 (0.06-1.07)	0.06	0.26 (0.08-0.87)	0.03
Chlamydia (N=9446)	10.6 (3.7)	16.4 (0.6)	0.61 (0.28-1.31)	0.2	1.17 (0.50-2.73)	0.7

SE: standard error. OR: unadjusted odds ratio. AOR: adjusted odds ratio, adjusted for age, race, gender, education, income, BMI, and immunosuppressive medication use. CI: confidence interval. STDs: sexually transmitted diseases. All estimates are weighted to represent the national population. Each analysis has a different number of subjects due to different age and gender cutoffs for viral studies in NHANES. Analysis of STDs included additional data from the 2013-2014 NHANES cycle available at the time of study.

**Table 3:** Associations of viral markers with comparator outcomes (gout, kidney stones, and hypertension), US adults ages 20-69 (N = 9483).

	%** with outcome (SE**%**)**	%** w/o outcome (SE**%**)**	**OR (95**%** CI)**	**OR ** **P-value**	**AOR (95**%** CI)**	**AOR ** **p-value**
**Gout**						
HSV 1 ab (N=5446)	66.1 (7.1)	58.1 (1.7)	1.41 (0.79-2.53)	0.2	1.35 (0.74-2.48)	0.3
HSV 2 ab (N=5446)	13.4 (3.7)	18.1 (0.8)	0.70 (0.37-1.34)	0.3	0.57 (0.24-1.35)	0.2
HPV oral PCR (N=8689)	11.0 (2.6)	7.4 (0.5)	1.54 (0.88-2.68)	0.1	1.12 (0.64-1.94)	0.7
HPV vaginal PCR (N=3474)	34.2 (9.1)	40.8 (1.3)	0.76 (0.33-1.71)	0.6	0.75 (0.33-1.71)	0.5
**Kidney Stones**						
HSV 1 ab (N=5441)	65.8 (3.4)	57.7 (1.7)	1.41 (1.09-1.83)	0.009	1.21 (0.88-1.65)	0.2
HSV 2 ab (N=5441)	22.5 (3.7)	17.8 (0.8)	1.34 (0.87-2.08)	0.2	1.20 (0.73-1.96)	0.5
HPV oral PCR (N=8680)	8.0 (1.0)	7.5 (0.5)	1.07 (0.85-1.36)	0.6	1.01 (0.78-1.31)	0.7
HPV vaginal PCR (N=3472)	39.3 (3.5)	40.8 (1.4)	0.94 (0.68-1.30)	0.7	0.94 (0.68-1.30)	0.7
**Hypertension**						
HSV 1 ab (N=5437)	67.1 (2.4)	56.6 (1.8)	1.67 (1.34-2.09)	<0.001	0.91 (0.66-1.24)	0.5
HSV 2 ab (N=5437)	26.7 (1.9)	16.5 (0.7)	1.92 (1.57-2.36)	<0.001	0.96 (0.78-1.19)	0.7
HPV oral PCR (N=8680)	8.0 (0.8)	7.4 (0.5)	1.10 (0.87-1.40)	0.4	1.17 (0.88-1.56)	0.3
HPV vaginal PCR (N=3470)	39.6 (2.7)	40.9 (1.4)	0.93 (0.74-1.17)	0.5	0.89 (0.67-1.18)	0.4

SE: standard error. OR: unadjusted odds ratio. AOR: adjusted odds ratio, adjusted for age, race, gender, education, income, BMI, and immunosuppressive medication use. CI: confidence interval. STDs: sexually transmitted diseases. Control conditions were chosen based on known metabolic etiology and low likelihood of association with viral infections, same data collection method, relatively high prevalence, and a similar demographic distribution. After adjustment, there was no association between positive viral studies and control outcomes, as predicted. All estimates are weighted to represent the national population. Each analysis has a different number of subjects due to different age and gender cutoffs for viral studies in NHANES.

## Data Availability

All data generated or analyzed during this study are included in this published article.

## Appendix

### A.

See Table S1.

### B.

See Table S2.

## Supplementary Materials

Supplementary Table 1 Full logistic regression models of arthritis with viral markers and adjustment variables.

Supplementary Table 2 Sensitivity analyses.
