# Supplementary material for: Population-Wide Associations between Common Viral Pathogens and Self-Reported Arthritis: NHANES 2009-2012
Source: Int J Rheumatol. 2018 Oct 1;2018:7684942. doi: 10.1155/2018/7684942 (PMC6188724; doi:10.1155/2018/7684942)
Supplement: Supplementary Table 1 — Full logistic regression models of arthritis with viral markers and adjustment variables. [file 7684942.f1.docx]

Table S1: Full logistic regression models of arthritis with viral markers and adjustment variables

|  | **Model 1**  **HSV 1 Ab**  **Adjusted OR**  **(95% CI)** | **Model 2**  **HSV 2 Ab**  **Adjusted OR**  **(95% CI)** | **Model 3**  **HPV oral PCR**  **Adjusted OR**  **(95% CI)** | **Model 4**  **HPV vaginal PCR**  **Adjusted OR**  **(95% CI)** |
| --- | --- | --- | --- | --- |
|  | 1.25 (0.96-1.62) | 1.48 (1.1-1.99) | 1.63 (1.17-2.28) | 1.35 (0.97-1.88) |
| **Age** | 1.09 (1.08-1.11) | 1.09 (1.08-1.11) | 1.08 (1.07-1.09) | 1.10 (1.08-1.12) |
| **Female gender** | 1.40 (1.09-1.81) | 1.35 (1.04-1.75) | 1.51 (1.30-1.76) | - |
| **Race**  Non-Hispanic Black | 1 (Ref.) | 1 (Ref.) | 1 (Ref.) | 1 (Ref.) |
| Non-Hispanic White | 1.73 (1.25-2.39) | 1.94 (1.38-2.72) | 1.23 (0.98-1.54) | 1.33 (0.97-1.83) |
| Hispanic | 0.69 (0.51-0.94) | 0.81 (0.59-1.10) | 0.67 (0.55-0.82) | 0.59 (0.42-0.82) |
| Other | 0.62 (0.36-1.04) | 0.70 (0.38-1.27) | 0.71 (0.53-0.95) | 0.76 (0.44-1.30) |
| **Education**  College and higher | 1 (Ref.) | 1 (Ref.) | 1 (Ref.) | 1 (Ref.) |
| GED/AA degree | 1.27 (0.91-1.77) | 1.24 (0.88-1.75) | 1.28 (1.04-1.57) | 1.80 (1.30-2.49) |
| Less than High school | 1.48 (0.91-2.40) | 1.48 (0.91-2.43) | 1.50 (1.18-1.91) | 1.85 (1.22-2.83) |
| **Annual HH income $** | 0.95 (0.92-0.97) | 0.95 (0.92-0.97) | 0.96 (0.94-0.98) | 0.95 (0.91-0.98) |
| **BMI** | 1.04 (1.03-1.06) | 1.04 (1.03-1.06) | 1.05 (1.04-1.06) | 1.05 (1.03-1.07) |
| **Immunosuppressive medication use** | 7.45 (3.33-16.68) | 7.38 (3.31-16.46) | 4.98 (2.63-9.41) | 6.31 (3.06-13.02) |

Table S1 Legend: HSV – herpes simplex virus. Ab - antibody. HPV – human papillomavirus. PCR – polymerase chain reaction. OR –odds ratio. CI – confidence interval. . GED – general educational development. AA – associate’s. HH – household. BMI – body mass index. Reference category not displayed for binary variables. All estimates are weighted to represent the national population.
