# Supplementary material for: Population-Wide Associations between Common Viral Pathogens and Self-Reported Arthritis: NHANES 2009-2012
Source: Int J Rheumatol. 2018 Oct 1;2018:7684942. doi: 10.1155/2018/7684942 (PMC6188724; doi:10.1155/2018/7684942)
Supplement: Supplementary Table 2 — Sensitivity analyses. [file 7684942.f2.docx]

Tables S2: Sensitivity analyses.

Table S2-1. Prevalence of positive viral markers by self-reported arthritis type.

|  | **No Arthritis** | **Osteo-arthritis** | **Rheumatoid Arthritis** | **Psoriatic Arthritis** | **Don’t Know** | **Other** |
| --- | --- | --- | --- | --- | --- | --- |
|  | **N = 7578** | **N = 550** | **N = 468** | **N = 21** | **N = 633** | **N = 230** |
| **Viral Markers** |  |  |  |  |  |  |
| HSV 1 ab, % (SE)** | 56.0 (1.4) | 67.8 (4.5) | 64.9 (5.6) | 82.3 (15.7) | 73.3 (5.2) | 65.9 (6.5) |
| HSV 2 ab, % (SE)*** | 15.7 (0.7) | 36.5 (4.4) | 32.7 (5.7) | 20.1 (15.8) | 28.7 (3.7) | 26.6 (5.6) |
| HPV oral PCR, % (SE)* | 6.9 (0.5) | 9.4 (2.0) | 9.1 (2.1) | 7.2 (6.6) | 11.7 (2.0) | 12.3 (3.3) |
| HPV vaginal PCR, % (SE) | 40.4 (1.3) | 34.3 (4.3) | 46.1 (5.5) | 26.6 (17.6) | 44.7 (6.6) | 50.4 (7.5) |

Table S2-1 Legend: * chi-square p value <0.05; ** chi-square p value <0.01; *** chi-square p value <0.001. The prevalence of HSV 1 and HSV 2 seropositivity, as well as HPV DNA positivity in oral rinse varied by type of arthritis reported by the study subjects. HSV 1 seropositivity was most frequent among subjects who reported psoriatic arthritis. HSV 2 seropositivity – among those reporting osteoarthritis, and HPV oral DNA positivity – among subjects who classified their arthritis as “other”. Please note that misclassification of arthritis type is highly likely by self-report.

Table S2-2. List of Lexicon immunosuppressive medications used in adjusted models.

| **Drug class** | **Medications** |
| --- | --- |
| Corticosteroids (non-topical) | Prednisone, prednisolone, dexamethasone, methylprednisolone, cortisone, hydrocortisone, budesonide |
| DMARDs | Methotrexate, leflunomude, hydroxychloroquine, sulfasalazine |
| Biologicals | Adalimumab, etanercept, infliximab, natalizumab, miscellaneous biologicals |
| Other immunosuppressives | Cyclophosphamide, azathioprine, cyclosporine, tacrolimus, sirolimus mycophenolate mofetil, mycophenolic acid |

Table S2-2 Legend: DMARDs – Disease-Modifying Antirheumatic Drugs. All unlisted biologicals were classified as miscellaneous in NHANES. Self-reported use of any immunosuppressive medication in the past 30 days was classified as positive.

Table S2-3. Association of viral markers with self-reported arthritis excluding participants taking immunosuppressive medications.

|  | **Not on immunosuppressives**  **(N = 9315)**  **AOR (95% CI)** | **AOR**  **P-value** | **Total adjusted estimates**  **(N = 9483)**  **AOR (95% CI)** | **AOR**  **p-value** |
| --- | --- | --- | --- | --- |
| **Viral Markers** |  |  |  |  |
| HSV 1 ab (N=5438) | 1.30 (0.98-1.72) | 0.07 | 1.25 (0.96-1.62) | 0.09 |
| HSV 2 ab (N=5438) | 1.45 (1.05-2.00) | 0.02 | 1.48 (1.10-1.99) | 0.009 |
| HPV oral PCR (N=8678) | 1.66 (1.20-2.30) | 0.002 | 1.63 (1.17-2.28) | 0.004 |
| HPV vaginal PCR (N=3469) | 1.38 (0.99-1.93) | 0.06 | 1.22 (0.90-1.66) | 0.2 |

Table S2-3 Legend: AOR – adjusted odds ratio, adjusted for age, race, gender, education, income, BMI. CI – confidence interval. All estimates are weighted to represent the national population. Each analysis has a different number of subjects due to different age and gender cutoffs for viral studies in NHANES.
